# 2017 Guidelines for Arterial Hypertension Management in Primary Health Care in Portuguese Language Countries

**DOI:** 10.5935/abc.20170165

**Published:** 2017-11

**Authors:** Gláucia Maria Moraes de Oliveira, Miguel Mendes, Marcus Vinícius Bolívar Malachias, João Morais, Osni Moreira Filho, Armando Serra Coelho, Daniel Pires Capingana, Vanda Azevedo, Irenita Soares, Alda Menete, Beatriz Ferreira, Miryan Bandeira dos Prazeres Cassandra Soares, Mário Fernandes

**Affiliations:** 1 Universidade Federal do Rio de Janeiro (UFRJ), Rio de Janeiro, RJ - Brazil; 2 Centro Hospitalar de Lisboa Ocidental, E.P.E. - Hospital de Santa Cruz, Carnaxide - Portugal; 3 Faculdade Ciências Médicas de Minas Gerais, Belo Horizonte, MG - Brazil; 4 Fundação Educacional Lucas Machado (FCMMG/FELUMA), Belo Horizonte, MG - Brazil; 5 Centro Hospitalar de Leiria - Hospital de Santo André, Leiria - Portugal; 6 Pontifícia Universidade Católica do Paraná, Curitiba, PR - Brazil; 7 Clínica Santos Dumont, Lisboa - Portugal; 8 Instituto Superior de Ciências de Saúde do Cuando Cubango de Angola - Angola; 9 Colégio da Especialidade de Cardiologia da Ordem dos Médicos de Cabo Verde - Cabo Verde; 10 Instituto do Coração de Moçambique - Moçambique; 11 Colégio da Especialidade de Cardiologia da Ordem dos Médicos de Moçambique - Moçambique; 12 Hospital Dr. Ayres de Menezes, São Tomé - São Tomé e Príncipe; 13 Hospital Américo Boavida, Luanda - Angola

**Keywords:** Hypertension / complications, Chronic Disease / mortality, Dyslipidemias, Obesity, Community of Portuguese-Speaking Countries

## Introduction

The World Health Organization (WHO) goal to reduce mortality due to chronic non-communicable diseases (CNCD) by 2% per year requires a huge effort from countries.^1-4^ This challenge for health professionals asks for a global political action on control of social measures, with cost-effective population interventions to reduce CNCD and their risk factors (RF). Health professionals should demand from their government the implementation of acceptable cost measures, such as tobacco cessation counseling, guidance on healthy feeding practices and need for regular physical exercise, systemic arterial hypertension (SAH) control, and promotion of teaching and updating activities in programs directed to those issues. Those measures would contribute with around 70% of the goal of 2% per year reduction in CNCD.^2,5^ Dyslipidemia, SAH and obesity are highly prevalent multifactorial diseases in Portuguese language countries (PLC).^5,6^ Systemic arterial hypertension is the major RF for complications, such as stroke, acute myocardial infarction and chronic kidney disease, corresponding in importance to dyslipidemia and obesity for the development of atherosclerotic diseases.^5,6^ In addition to their significant epidemiological impact, the non-pharmacological treatment of those cardiovascular RF plays a relevant economic role in the expenditures of the Ministries of Health, Social Security and Economy, because those affections are major causes directly or indirectly involved with absenteeism in the workplace. There is evidence that preventive actions are more promising in the primary health care setting.

The number of adults with SAH increased from 594 million in 1975 to 1.13 billion in 2015, being 597 million men and 529 million women. That increase might be due to both population aging and increase in number.^6^ When analyzing the trends in blood pressure (BP) levels of 19.1 million adults from several population studies in the past four decades (1975-2015), the elevated levels shifted from high-socioeconomic-level countries to low-intermediate-socioeconomic-level countries of South Asia and Sub-Saharan Africa. However, BP levels remain high in Eastern and Central Europe and Latin America.^6^

Several trends were identified when analyzing the proportional mortality and percentage change in the mortality rates due to hypertensive diseases and their outcomes, ischemic heart diseases (IHD) and stroke, in the PLC from 1990 to 2015 (Table 1). The highest proportional mortality rates due to hypertensive diseases were observed in Brazil, Mozambique and Angola. Portugal had the highest human development index (HDI) in 2015 and the highest mortality due to stroke.^7-9^ The reduced access, around 50-65%, to essential pharmacological treatment in low-and low-intermediate-socioeconomic-level countries might have contributed to those results. In addition, in 40% of those countries there is less than 1 physician per 1000 in habitants, and a small number of hospital beds for the care of the uncontrolled-SAH-related outcomes.^7^ Thus, joint actions to implement primary prevention measures can reduce the outcomes related to hypertensive disease, especially IHD and stroke. It is mandatory to ensure the implementation of guidelines for the management of SAH via a continuous process, involving educational actions, lifestyle changes and guaranteed access to pharmacological treatment.

**Table 1:** Proportional mortality and annual percentage of change in mortality rates in both sexes, all ages, from 1990 to 2015, due to hypertensive disease, ischemic heart disease and stroke, in addition to human development index (HDI) and population in 2015

Countries	Hypertensive disease	Ischemic heart disease	Stroke	HDI 2015	Population 2015*
	Proportional mortality (annual % change in mortality rates)				
Brazil	1.77 (+1.79)	14.44 (+0.44)	10.61 (+0.12)	0.754*	205,002,000
Mozambique	1.46 (+0.27)	3.84 (+1.25)	5.37 (+0.52)	0.418*	25,727,911
Angola	1.28 (-0.97)	4.65 (-0.96)	5.35 (-1.09)	0.533*	25,789,024
Portugal	1.08 (+1.20)	12.71 (-1.32)	14.96 (-2.32)	0.843*	10,374,822
Guinea-Bissau	0.53 (-0.43)	4.87 (+0.25)	5.07 (+0.22)	0.424*	1,844,000
East Timor	1.33 (+0.38)	11.84 (+1.16)	10.02 (+0.57)	0.605*	1,212,107
Macao	NA	NA	NA	0.566#	642,900
Cape Verde	0.75 (-0.62)	11.74 (+1.34)	13.74 (-0.18)	0.648*	524,833
Saint Thomas and Prince	0.44 (-0.55)	8.18 (-0.41)	10.22 (-0.18)	0.574*	190,000

* last year available - 2015,

# last year available - 2014, NA: not available. Source:^7-9^

### Diagnosis and classification

The risk resulting from high BP levels increases with age, and every 2-mmHg elevation is associated with a 7% and a 10% increase in the risk of death due to IHD and stroke, respectively.^2^ At the medical office, BP can be assessed by use of either the automated or auscultatory method, being elevated when systolic BP (SBP) ≥ 140 mm Hg and/or diastolic BP (DBP) ≥ 90 mm Hg, at least on two occasions.

**Figure 1:**
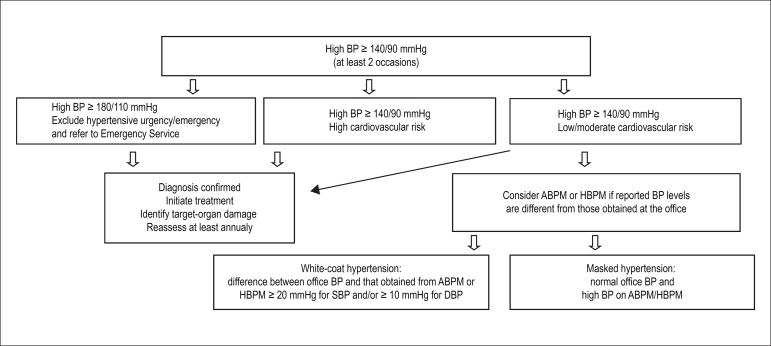
Flowchart for the diagnosis of arterial hypertension. BP: blood pressure; ABPM: ambulatory BP monitoring; HBPM: home BP monitoring; SBP: systolic BP; DBP: diastolic BP.

The diagnosis of SAH is based on the measurement at the doctor's office of two or more high BP values on at least two occasions. The classification of BP according to measurements taken at the medical office, for individuals older than 18 years, is shown in Table 2. Ambulatory BP monitoring for 24 hours (ABPM) or home BP monitoring (HBPM) can help in the diagnosis of white-coat hypertension (WCH) and masked hypertension (MH). The WCH relates to the difference between BP measured at the office (high) and that measured with ABPM or HBPM (normal). In MH, the situation is the opposite (Figure 1). In view of the suspicion of WCH and MH, ABPM is mandatory, and may be replaced by HBPM in communities where ABPM is not available. Figure 1 shows the flowchart for the diagnosis of SAH.

**Table 2:** Blood pressure classification according to measurements taken at the office for individuals older than 18 years

Classification	SBP (mm Hg)	DBP (mm Hg)
Normal	≤ 120	≤ 80
Prehypertension	121 – 139	81 – 89
Stage 1 hypertension	140 – 159	90 – 99
Stage 2 hypertension	160 – 179	100 – 109
Stage 3 hypertension	≥ 180	≥ 110

When SBP and DBP are in different categories, the highest should be used to classify BP.

Systolic hypertension is considered isolated if SBP ≥ 140 mm Hg and DBP < 90 mm Hg, and it should be classified into stages 1, 2 and 3. SBP: systolic blood pressure; DBP: diastolic blood pressure. Source: 7th Brazilian guideline for arterial hypertension management, 2016.^1^

The ABPM enables the identification of circadian BP changes, especially those related to sleep. In ABPM, BP is considered increased when BP in 24 hours ≥ 130/80 mmHg, ranging from wakefulness ≥ 135/85 mm Hg to sleep ≥ 120/70 mmHg. For HBPM, BP is considered elevated when ≥ 135/85 mmHg.^1^

### Recommended technique for measuring blood pressure

Initially the patients should be informed about the procedure, and the steps on Table 3 should be followed.^3,10,11^ Blood pressure should be measured by all health professionals on every clinical assessment and at least once a year.

**Table 3:** Recommended technique for measuring office blood pressure by using the auscultatory method

•	BP should be measured with a validated, calibrated and accurate sphygmomanometer, with cuff size adequate to arm circumference (according to the manufacturer's recommendation): usually cuff width close to 40% and cuff length covering 80-100% of arm circumference.
•	The cuff should be placed snugly, 2-3 cm above the cubital fossa, with its compressive part centralized on the brachial artery, and the arm supported at heart level.
•	The patient should rest at a calm environment for 5 minutes, sitting in a chair with back supported, legs uncrossed and feet on the floor. The patient should be relaxed, having neither exercised in the previous 30 minutes, nor consumed tobacco, alcohol or energetic foods (including coffee) in the previous 1 hour.
•	In addition, BP will be measured after 2 minutes in the supine position with the arm supported, especially for diabetics and the elderly, and when orthostatic hypotension is suspected. It is worth noting that measuring BP in the sitting position will be useful for therapeutic decision-making, while that in the orthostatic position, for treatment changes in case of orthostatic hypotension.
•	The cuff should be inflated rapidly up to 30 mm Hg above the level the radial pulse can no longer be palpated, and then deflated at approximately 2 mm Hg/beat. SBP will be determined by auscultation of the first sound (Korotkoff phase I), and DBP, by disappearance of the sounds (Korotkoff phase V). If the heart beats persist until level zero, determine DBP on the muffling of sounds (Korotkoff phase IV).
•	The first reading should be discarded, and two sequential readings in both members should be taken, the highest one being recorded. If arrhythmia is present, more measurements should be taken to determine mean BP.
•	Record the BP reading obtained for the patient. Reassess BP levels at least monthly until control is achieved, and then every 3 months.

BP: blood pressure; SBP: systolic blood pressure; DBP: diastolic blood pressure.

### Clinical assessment and risk stratification

Complementary assessment is aimed at detecting target-organ damage (TOD), aiding cardiovascular risk stratification and identifying signs of secondary SAH. Table 4 shows the recommended complementary tests (routine and for specific populations).

**Table 4:** Recommended complementary tests (routine and for specific populations)

Routine tests for all hypertensive patients	
Urinalysis	Fasting glycemia and HbA1c
eGFR	Total cholesterol, HDL-C and serum triglycerides
Conventional ECG	Serum levels of creatinine, potassium and uric acid
**Recommended tests to search for TOD in specific populations**	
Chest X ray	Clinical suspicion of cardiac and/or pulmonary impairment. Aortic dilatation or aneurysm (if echocardiogram is not available). Suspicion of aorta coarctation.
Echocardiogram	Evidence of LVH on ECG or patients with clinically suspected HF. LVH = LV mass corrected for BS ≥ 116 g/m^2^ (men) or 96 g/m^2^ (women)
Albuminuria	Diabetic hypertensive patients, with metabolic syndrome or at least two RF. Normal values < 30 mg/24h.
Carotid US	Carotid murmur, CbVD signs, atherosclerotic disease in other sites. IMT values > 0.9 mm and/or atherosclerotic plaques.
Renal US or Doppler	Patients with abdominal masses or abdominal murmurs.
Exercise test	Suspicion or family history of CAD, DM.
Brain MRI	Patients with cognitive disorders and dementia. Detection of silent infarctions and micro hemorrhages.

HbA1c: glycated hemoglobin; eGFR: estimated glomerular filtration rate; TOD: target-organ damage; ECG: electrocardiogram; LVH: left ventricular hypertrophy; HF: heart failure; LV: left ventricular; BS: body surface; RF: risk factors; US: ultrasonography; CbVD: cerebrovascular disease; IMT: intima-media thickness; CAD: coronary artery disease; DM: diabetes mellitus; MRI: magnetic resonance imaging.

- Target-organ damage should be investigated with the complementary tests shown in Table 4, in addition to the following exams:
- Left ventricular hypertrophy, assessed on electrocardiogram: Sokolow-Lyon index [S in V1 + R in V5 or V6 (whichever is larger)] > 35 mm; RaVL > 1.1 mV; Cornell index [S in V3 + R in aVL > 28 mm (men), and S in V3 + R in aVL > 20 mm (women)]; or on echocardiogram: left ventricular mass index ≥ 116 g/m^2^ (men), and ≥ 96 g/m^2^ (women);

Atherosclerotic disease in other sites and chronic kidney disease ≥ stage 3 [estimated glomerular filtration rate (eGFR) > 60 mL/min/1.73 m^2^] (Table 5).

**Table 5:** Stratification based on risk factors, target-organ damage and cardiovascular or kidney disease

	SBP 130-139 or DBP 85-89	Stage 1 SAH SBP 140-159 or DBP 90-99	Stage 2 SAH SBP 160-179 or DBP 100-109	Stage 3 SAH SBP ≥ 180 or DBP ≥ 110
No risk factor	No additional risk	Low risk	Intermediate risk	High risk
1-2 risk factors	Low risk	Intermediate risk	High risk	High risk
≥ 3 risk factors	Intermediate risk	High risk	High risk	High risk
Presence of TOD, CVD, CKD or DM	High risk	High risk	High risk	High risk

BP: blood pressure; SBP: systolic blood pressure; DBP: diastolic blood pressure; SAH: systemic arterial hypertension; TOD: target-organ damage; CVD: cardiovascular disease; CKD: chronic kidney disease; DM: diabetes mellitus. Source: 7th Brazilian guideline for arterial hypertension management, 2016.^1^

Risk stratification should consider the classical RF, relating them to BP levels as shown in Table 5.

The following risk factors are considered:

- male sex and age (men > 55 years and women > 65 years);
- smoking habit, dyslipidemia (triglycerides > 150 mg/dL; LDL-C > 100 mg/dL; HDL-C < 40 mg/dL), obesity (body mass index ≥ 30 kg/m^2^), abdominal obesity (abdominal circumference > 102 cm for men, and > 88 cm for women), diabetes mellitus, abnormal oral glucose tolerance test or fasting glycemia of 102-125 mg/dL, and family history of premature cardiovascular disease (men < 55 years, and women < 65 years).

### Treatment

Blood pressure reduction is followed by a significant cardiovascular risk reduction, which is higher in individuals at high cardiovascular risk, with a relative residual risk reduction in the other individuals.^2,11^ Non-pharmacological therapy with changes in lifestyle (CLS) should be initially implemented for all stages of SAH and for individuals with BP of 135-139/85-89 mmHg (Table 6). For stage 1 hypertensives at low or intermediate cardiovascular risk, management can start with CLS, and 3 to 6 months can be waited before deciding to start pharmacological treatment. For the other stages, antihypertensive agents should be initiated as soon as the diagnosis is established.

**Table 6:** Recommendations for the non-pharmacological treatment of arterial hypertension

Measure	Recommendations	
Body weight control	Maintain BMI < 25 kg/m^2^ up to 65 years of age;	
	Maintain BMI < 27 kg/m^2^ after 65 years of age;	
	Maintain AC < 88 cm for women and < 102 cm for men.	
Dietary pattern	Adopt a diet rich in fruits and vegetables, with a reduced amount of saturated fat.	
	The DASH (Dietary Approach to Stop Hypertension) diet, with 2100 kcal/day as originally proposed, is the most used:	
	Fruits (portions/day)	4-5
	Vegetables (portions/day)	4-5
	Milk and dairy products < 1% fat (portions/day)	2-3
	Lean meat, fish and poultry (g/day)	< 180
	Oils and fats (portions/day)	2-3
	Seeds and nuts (portions/week)	4-5
	Added sugars (portions/week)	< 5
	Salt (portion/day)	~ 6 g (3000 mg of sodium)
	Whole grains (portions/day)	6-8
Moderate alcohol consumption	Limit daily alcohol consumption to 1 dose for women and low-weight individuals, and 2 doses for men.	
Physical activity	**For all hypertensives – population recommendation – physical activity practice**	
	Moderate, continuous (1 x 30 min) or cumulative (2 x 15 min or 3 x 10 min) physical activity (similar to walking): at least 30 min/day, 5 to 7 days/week.	
	**Aerobic training**	
	At least 3 times/week (ideally 5 times/week), minimum of 30 min (ideally 40 to 50 min); Several modalities: walking, running, dancing, swimming; Moderate intensity defined as: higher intensity that still allows talking (no breathlessness), and sensation of mild to moderate tiredness; Maintain training heart rate (THR) between the lower and upper THR calculated as follows: Lower THR = (maximum HR – resting HR) x 0.5 + resting HR*; upper THR = (maximum HR – resting HR) x 0.7+ resting HR* Ideally, the HR used to calculate the intensity of the aerobic training should be determined on a maximum exercise test, with patients on their usual medication. *Maximum HR: obtained either on a maximum exercise test with regular medications, or by calculating maximum HR estimated according to age (220 - age; not to be used for individuals with heart disease or hypertensives on beta-blockers or nondihydropyridine calcium channel blockers). Resting HR: measured after a 5-minute rest, lying down.	
	**Resistance training**	
	2 - 3 times/week, 8 - 10 exercises for the large muscle groups, prioritizing unilateral execution, when possible; 1 - 3 sets with 10 - 15 repetitions up to moderate fatigue (reducing the movement velocity and avoiding apnea, exhaling during contraction and inhaling when returning to the initial position); Long passive pauses: 90 - 120 s.	

BMI: body mass index; AC: abdominal circumference. Source: Adapted from the 7th Brazilian guideline for arterial hypertension management, 2016.^1^

**Figure 2:**
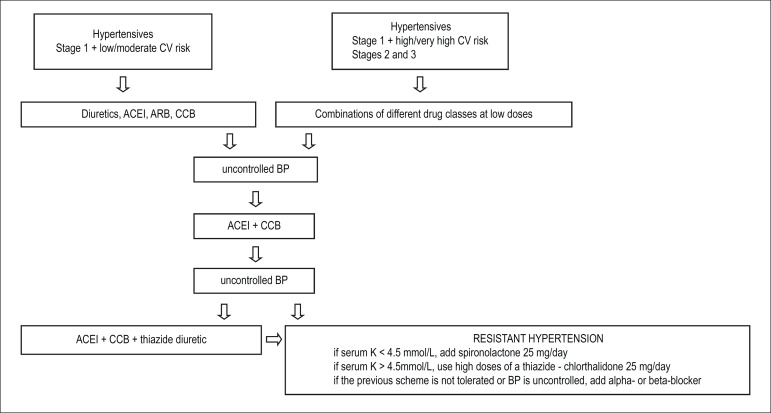
Flowchart for the treatment of arterial hypertension. (adapted from Malachias et al^1^) CV: cardiovascular; BP: blood pressure; ACEI: angiotensin-converting-enzyme inhibitor; ARB: angiotensin-receptor blocker; CCB: calcium-channel blocker.

A BP target lower than 130/80 mm Hg is recommended for patients at high cardiovascular risk, including those with diabetes mellitus, and lower than 140/90 mm Hg for stage 3 hypertensives. For patients with coronary artery disease, BP should not be lower than 120/70 mm Hg because of the risk of coronary hypoperfusion, myocardial damage and cardiovascular events. For elderly hypertensives ≥ 80 years, BP levels should be lower than 145/85 mm Hg. Special attention should be paid to patients with dark skin phenotype who will benefit more from the use of calcium-channel blockers.^12-14^ Figure 2 shows the pharmacological approach to SAH.

When angiotensin-converting enzyme inhibitors (ACEI) are not tolerated, they should be replaced with low-cost angiotensin-receptor blockers (ARB). Beta-blockers should be considered for young individuals intolerant to ACEI and ARB, lactating women, individuals with increased adrenergic tone, and those with IHD or heart failure (HF). In case of intolerance to calcium-channel blockers (CCB) because of edema, or HF or suspected HF, diuretics can be used: thiazide diuretics (chlorthalidone - 12.5-25 mg 1X day; indapamide - 1.5-2.5 mg 1X day). Individuals with dark skin phenotype should have ARBs rather than ACEIs for pharmacological combinations.^2,11-14^

Approximately two thirds of the patients will need combinations of at least two drugs to control BP. The advantage of the association is the synergism of different mechanisms of action, with dose reduction and consequent decrease in adverse effects, in addition to higher therapeutic adherence.

There is no preference for a therapeutic class of drug to treat a hypertensive patient with a previous stroke, but a BP lower than 130/80 mm Hg should be targeted.

Table 7 depicts the clinical situations with indication for or contraindication to specific drugs. For chronic kidney disease, ACEI and ARB reduce albuminuria, and thiazide diuretics are used for stages 1 to 3, while loop diuretics, for stages 4 and 5.^2,11-14^

**Table 7:** Clinical situations with indication for or contraindication to specific drugs

Drugs with specific indication	
Clinical situation	Initial therapy indicated
Heart failure	ACEI/ARB, diuretics and BB
AMI, angina pectoris, percutaneous or surgical myocardial revascularization	ACEI/ARB, BB, ASA, statins
Diabetes mellitus	Thiazide diuretics, ACEI/CCB, BB
Chronic renal failure	ACEI/ARB, loop diuretics
Metabolic syndrome	CCB, ACEI/ARB
Aortic aneurysm	BB
Peripheral arterial disease	ACEI, CCB
Pregnancy	Methyldopa, CCB
**Contraindicated drugs**	
Clinical situation	Contraindicated therapy
Asthma and chronic bronchitis	Non-cardioselective BB
Pregnancy	ACEI, ARB
AV block	BB, nondihydropyridine CCB
Gout	Diuretics
Bilateral stenosis of the renal artery	ACEI, ARB

ACEI: angiotensin-converting-enzyme inhibitor; ARB: angiotensin-receptor blocker; CCB: calcium-channel blocker; BB: beta-blockers; AMI: acute myocardial infarction; ASA: acetylsalicylic acid; AV: atrioventricular.

* ACEI and ARB should not be associated, because of the ONTARGET study. Adapted from^2,4^

### Arterial hypertension in pregnancy

Pregnant women with uncomplicated chronic hypertension should have BP levels lower than 150/100 mmHg, but DBP should not be < 80 mmHg.^1,2,11-14^ The use of ACEI and ARB is contraindicated during pregnancy, and atenolol and prazosin should be avoided. Methyldopa, beta-blockers (except atenolol), hydralazine and CCBs (nifedipine, amlodipine and verapamil) can be safely used.^2,11-14^

In chronic gestational hypertension with TOD, BP levels should be maintained under 140/90 mmHg, and the pregnant woman should be referred to a specialist for proper care during delivery and to avoid teratogenicity. Delivery should not be hastened if BP < 160/110 mmHg (with or without anti-hypertensive drugs) up to the 37^th^ week. The fetal growth and amount of amniotic fluid should be monitored with ultrasonography between the 28^th^ and 30^th^ weeks and between the 32^nd^ and 34^th^ weeks, and with umbilical artery Doppler. During delivery, BP levels should be monitored continuously.^1,2,12-14^ During the puerperium period, BP levels should be maintained under 140/90 mmHg, preferably with the following drugs, whose use is safe during lactation: hydrochlorothiazide, spironolactone, alpha-methyldopa, propranolol, hydralazine, minoxidil, verapamil, nifedipine, nimodipine, nitrendipine, benazepril, captopril and enalapril.^1,2,12-15^

Preeclampsia (PE) is defined by the presence of SAH after the 20^th^ gestational week, associated with significant proteinuria or presence of headache, blurred vision, abdominal pain, low platelet count (< 100,000/mm^3^), elevation of liver enzymes (twice the baseline level), kidney impairment (creatinine > 1.1 mg/dL or twice the baseline level), pulmonary edema, visual or cerebral disorders and scotomas. Eclampsia occurs when grand mal seizure associates with PE. The use of magnesium sulfate is recommended to prevent and treat eclampsia, at an attack dose of 4-6 g IV for 10-20 minutes, followed by infusion of 1-3 g/h, usually for 24 hours after the seizure. In case of relapse, 2-4 g IV can be administered. The use of corticosteroids, IV anti-hypertensives (hydralazine, labetalol) and blood volume expansion are recommended. Patients should be admitted to the intensive care unit.^1,2,11-15^

Table 8 lists the reasons for not achieving proper BP control. It is worth noting the importance of ruling pseudoresistance out (WCH).

**Table 8:** Possible reasons of not achieving proper blood pressure control

•	Inadequate adherence to medications, diet, physical activity practice, and consumption of salt, tobacco and alcohol.
•	Associated conditions: overweight and obesity, obstructive sleep apnea, chronic pain, blood volume overload, chronic kidney disease, thyroid disease.
•	Drug interaction: nonsteroidal anti-inflammatory drugs, corticosteroids, anabolic steroids, sympathomimetic drugs, decongestants, amphetamine, erythropoietin, cyclosporine, tacrolimus, licorice, monoamine oxidase inhibitors, serotonin and norepinephrine reuptake inhibitors.
•	Suboptimal therapeutic regimen, low doses of drugs, inappropriate combinations of anti-hypertensive drugs, renal sodium retention (pseudotolerance).
•	Secondary hypertension: renovascular disease, primary hyperaldosteronism, pheochromocytoma.

Source: Leung et al.^11^

### Secondary arterial hypertension

The prevalence of secondary SAH in the hypertensive population is around 3-5%. The most common cause of secondary SAH is renal parenchymal disease, responsible for 2-5% of the SAH cases. The adrenal causes of SAH and pheochromocytoma occur in less than 1% of all cases of SAH. However, 80% of the patients with Cushing's syndrome have SAH. Physicians must keep a high level of clinical suspicion when managing hypertensives of difficult control. Table 9 lists the clinical findings of the major etiologies of secondary SAH, associating them with the complementary tests that should be used to establish the diagnosis.

**Table 9:** Causes of secondary SAH, signs and complementary diagnostic tests

Clinical findings	Diagnostic suspicion	Additional studies
Snoring, daytime sleepiness, MS	OSAHS	Berlin questionnaire, polysomnography or home respiratory polygraphy with at least 5 episodes of apnea and/or hypopnea per sleep hour
RAH and/or hypopotassemia (not necessary) and/or adrenal nodule	Primary hyperaldosteronism (adrenal hyperplasia or adenoma)	Measurements of aldosterone (> 15 ng/dL) and plasma renin activity/concentration; aldosterone/renin > 30. Confirmatory tests (furosemide and captopril). Imaging tests: thin-sliced CT or MRI
Edema, anorexia, fatigue, high creatinine and urea, urine sediment changes	Kidney parenchymal disease	Urinalysis, eGFR calculation, renal US, search for albuminuria/proteinuria
Abdominal murmur, sudden APE, renal function changes due to drugs that block the RAAS	Renovascular disease	Renal Doppler US and/or renogram, angiography via MRI or CT, renal arteriography
Absent or decreased femoral pulses, decreased blood pressure in the lower limbs, chest X ray changes	Coarctation of the aorta	Echocardiogram and/or chest angiography via CT
Weight gain, decreased libido, fatigue, hirsutism, amenorrhea, 'moon face', 'buffalo hump', purple striae, central obesity, hypopotassemia	Cushing's syndrome (hyperplasia, adenoma and excessive production of ACTH)	Salivary cortisol, 24-h urine free cortisol and suppression test: morning cortisol (8h) and 8 hours after administration of dexamethasone (1 mg) at 12PM. MRI
Paroxysmal AH with headache, sweating and palpitations	Pheochromocytoma	Free plasma metanephrines, plasma catecholamines and urine metanephrines. CT and MRI
Fatigue, weight gain, hair loss, DAH, muscle weakness	Hypothyroidism (20%)	TSH and free T4
Intolerance to heat, weight loss, palpitations, exophthalmos, hyperthermia, hyperreflexia, tremors, tachycardia	Hyperthyroidism	TSH and free T4
Renal lithiasis, osteoporosis, depression, lethargy, muscle weakness or spasms, thirst, polyuria	Hyperparathyroidism (hyperplasia or adenoma)	Plasma calcium and PTH
Headache, fatigue, visual disorders, enlarged hands, feet and tongue	Acromegaly	IGF-1 and GH levels at baseline and during oral glucose tolerance test

MS: metabolic syndrome; OSAHS: obstructive sleep apnea-hypopnea syndrome; RAH: resistant arterial hypertension; CT: computed tomography; MRI: magnetic resonance imaging; eGFR: estimated glomerular filtration rate; US: ultrasonography; APE: acute pulmonary edema; RAAS: renin-angiotensin-aldosterone system; ACTH: adrenocorticotropin; AH: arterial hypertension; DAH: diastolic arterial hypertension; TSH: thyroid stimulating hormone; PTH: parathormone; IGF-1: insulin-like growth factor type 1; GH: growth hormone. Source: Malachias et al.^1^

Similarly to CNCD, lifelong adherence to the SAH treatment is poor. In the first year, 40% of the patients quit regular treatment, which prevent them from profiting from a reduction in both TOD and cardiovascular events, such as myocardial infarction and stroke. The following factors are related to non-adherence to treatment: adverse effects, number of daily doses and drug tolerance. Fixed drug combinations increase adherence by enabling better individual adequacy, reducing the likelihood of irregular use of daily doses. The involvement of patients and families, as well as a multidisciplinary approach enhance adherence to treatment. The use of interactive apps that increase the participation of patients in BP control is suggested to encourage their persistence and regular medication use.^16^
